# Using Memristors for Robust Local Learning of Hardware Restricted Boltzmann Machines

**DOI:** 10.1038/s41598-018-38181-3

**Published:** 2019-02-12

**Authors:** Maxence Ernoult, Julie Grollier, Damien Querlioz

**Affiliations:** 1 Unite Mixte de Physique CNRS/Thales and Univ. Paris Sud, 91767 Palaiseau, France; 2 Centre de Nanosciences et de Nanotechnologies, Univ. Paris-Sud, CNRS, 91405 Orsay, France; 3 Sorbonne Université, 75006 Paris, France

## Abstract

One of the biggest stakes in nanoelectronics today is to meet the needs of Artificial Intelligence by designing hardware neural networks which, by fusing computation and memory, process and learn from data with limited energy. For this purpose, memristive devices are excellent candidates to emulate synapses. A challenge, however, is to map existing learning algorithms onto a chip: for a physical implementation, a learning rule should ideally be tolerant to the typical intrinsic imperfections of such memristive devices, and local. Restricted Boltzmann Machines (RBM), for their local learning rule and inherent tolerance to stochasticity, comply with both of these constraints and constitute a highly attractive algorithm towards achieving memristor-based Deep Learning. On simulation grounds, this work gives insights into designing simple memristive devices programming protocols to train on chip Boltzmann Machines. Among other RBM-based neural networks, we advocate using a Discriminative RBM, with two hardware-oriented adaptations. We propose a pulse width selection scheme based on the sign of two successive weight updates, and show that it removes the constraint to precisely tune the initial programming pulse width as a hyperparameter. We also propose to evaluate the weight update requested by the algorithm across several samples and stochastic realizations. We show that this strategy brings a partial immunity against the most severe memristive device imperfections such as the non-linearity and the stochasticity of the conductance updates, as well as device-to-device variability.

## Introduction

Fast progress in machine learning and big data processing make conventional electronics hardware unable to cope with it in the long run, and calls for breakthrough in artificial intelligence hardware design^1^. Novel nanoelectronic devices, such as memristive devices, or resistive memories, are particularly exciting in this regard, as they can emulate synapses when arranged into crossbar arrays with interconnecting transistors acting as neurons^2–5^. This can lead to hardware neural networks, with outstanding energy efficiency: they associate computation and memory very closely, whereas exchanges between computation and memory is the dominant source of energy consumption in conventional architectures^6^.

Such hardware neural networks can be trained *ex situ*: the synaptic weights are optimally determined on conventional central or graphical processing units, and then transferred onto memristive hardware^7,8^. Nevertheless, the most exciting applications could come from systems with a capability of learning. However, such *in situ* learning comes with two major challenges. First, programming the conductance of memristive device very precisely is difficult, due to well-known memristive device imperfections, such as non-linear conductance response, cycle-to-cycle and device-to-device variability^9^. In the case of *ex situ* learning, this difficulty can be avoided by using complex tuning protocols^7,10^. But in the case of *in situ* learning, such tuning protocols cannot be used as devices need to be reprogrammed repeatedly throughout learning.

The second challenge of *in situ* learning is the non-locality of most neural network learning rules. This is the case of backpropagation, the most widely used learning rule for neural networks in machine learning^11^: the weight update depends on synapses and neurons from other layers. Conversely, a local learning rule calls for a weight update which solely depends on the two neurons connected to this weight: from a hardware viewpoint, the conductance of a memristive device is simply programmed by the voltage difference between the pre- and post- synaptic neurons. For this reason, although its theoretical implementation with memristive devices has been extensively studied^12–18^, most demonstrations of memristive *in situ* learning hardware is single layer, when backpropagation becomes local^7,19^.

In this work, we investigate the possibility to perform *in situ* learning circumventing these two challenges entirely. For this purpose, we propose implementing variations of Restricted Boltzmann Machines (RBMs) that allow *in situ* learning with a local learning rule, and where memristive device programming can be achieved in a very simple way. RBMs are stochastic neural networks, whose basic principles have a remote inspiration from statistical physics, and which have found recent applications in pattern detection^20,21^. In software, RBMs often underperform with regards to the most sophisticated deterministic neural networks on benchmark data sets^22^. However, they appear extremely attractive with regards to our two challenges. They can indeed be trained with Contrastive Divergence^23^, a spatially local learning rule. Also, their intrinsically stochastic nature suggests that they could be appropriate to learn in an approximate setting. Existing works on memristive RBMs^24–28^ mainly focused on the CMOS circuitry to implement the neurons^24,26^, matrix multiplication and summation^26^, Gibbs sampling^28^, neuron value centering when adding depth^27^. In this study, we perform simulations and propose methods for achieving *in situ* learning. We focus in particular on the impact of the conductance update physics, and of the tuning of hyperparameters, a critical question for *in situ* learning.

We first introduce a baseline memristor-based gradient descent algorithm taking only the sign of the gradient into account. We use this algorithm to train the three most encountered RBM-based architectures in the neuromorphic literature on the hand-written digit classification task (MNIST^29^) with typical values of the device parameters to identify the most relevant algorithm. In the second part of the paper, we show that reducing the variance of the gradient estimate provided by Contrastive Divergence improves the performance of the RBM with non-linear devices. Pointing out the necessity to hand-tune the pulse width in the baseline algorithm, we come up with a programming pulse width selection based on the sign of two consecutive weight updates inspired from Resilient Propagation^30–32^ which enlarges the range of eligible pulse widths by up to two decades. Finally, we combine the two techniques introduced and analyze the effect of variability on the RBM. We conclude by lessons taught by these results.

## Results

### Memristor model used and associated learning algorithm

All the simulations presented in this paper have been carried out at a level which highlights the effects of the weight update physics and the learning rules it enables on the different neural network architectures introduced thereafter.

The following model^33^ for the memristive devices was used:

1 $$\frac{dG(t)}{dt}=\{\begin{array}{ll}{C}_{p}\,\exp \,(-{\beta }_{p}\frac{G(t)-{G}_{min}}{{G}_{max}-{G}_{min}}) & ({\rm{potentiation}})\\ -{C}_{d}\,\exp \,(-{\beta }_{d}\frac{{G}_{max}-G(t)}{{G}_{max}-{G}_{min}}) & ({\rm{depression}})\end{array},$$

applying Eq. (1) between *t*_0_ and *t*_0_ + Δ*t* yields the effective conductance update (whose explicit form is shown in the Methods):

2 $$G({t}_{0}+{\rm{\Delta }}t)=G({t}_{0})+{\int }_{{t}_{0}}^{{t}_{0}+\Delta t}\frac{dG(t)}{dt}dt,$$

*G*(*t*) denotes the current conductance at time *t* of the device, with *G*_*max*_ and *G*_*min*_ being the maximal and minimal conductance, labels *p* and *d* referring to potentiation and depression respectively. Δ*t* appearing in Eq. (2) defines the programming pulse width. Note that our memristor model implicitly takes into account the number of pulses applied to the device: it treats equally a programming pulse of width Δ*t* or n programming pulses of width Δ*t*/*n*. We also introduce Δ*t*_*max*_ as the pulse width that is required to bring the conductance from *G*_*min*_ to *G*_*max*_. *C*_*p*_ and *C*_*d*_, which encode the amplitude of the voltage difference applied to the device, is fixed to ensure this last condition (see Methods for details). *β*_*p*_ and *β*_*d*_ model the dependence of the conductance update with the current conductance, namely the non-linearity of the device so that if *β*_*p*_ = 0, *dG*/*dt* is constant for potentiation. This model can be used to describe real memristive devices^2,33–35^. Our model is similar in form to existing model^36^, as further discussed in the Methods.

**Figure 1:**
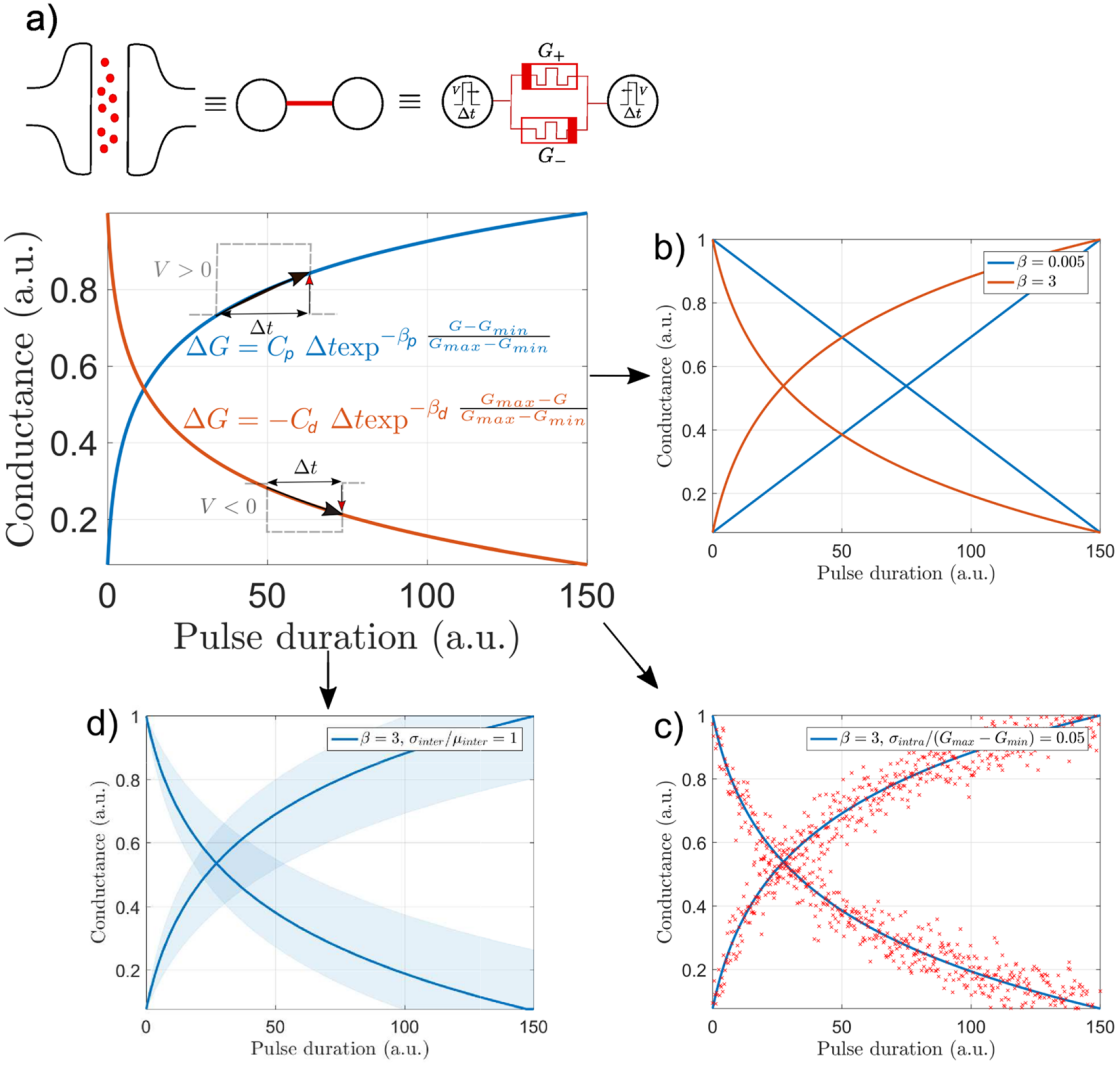
(**a**) Each weight is implemented with two memristors, i.e. *W* = *G*_+_  − *G*_−_, conductance updates follow the memristor characteristic dictated by Eq.(1). (**b**–**d**) Illustration of the memristive imperfections taken into account: (**b**) non-linearity (conductance dependent update), (**c**) cycle-to-cycle variability, (**d**) device-to-device variability (from left to right).

In our work, we assume that each model parameter or weight *W* is carried by two memristive devices of conductance *G*_+_ and *G*_−_, so that *W* = *G*_+_ − *G*_−_^12^ - see Fig. 1. In the light of these notations and for most of our simulations, we assumed that the non-linear parameter *β* and the multiplicative factor *C* were the same not only between two devices of the same synaptic pair, but also for potentiation and depression - in the absence of device variation, see below. Depending on the technology used, *G*_+_ and *G*_−_ can only be increased^12,37^, which our simulation framework can handle. The learning algorithm that we used proceeds by performing gradient descent on an objective function *J*. Proper standard gradient descent scheme prescribes the following learning rule:

3 $$W\leftarrow W-\alpha \nabla J(W),$$

where *α* and ∇*J*(*w*) are the learning rate and the gradient of the objective function respectively. However, incrementing precisely *W* = *G*_+_  − *G*_−_ of the amount *α*∇*J*(*w*) along the memristor characteristics Eq. (1) is extremely impractical: it requires to temporarily store the gradient value, read out the weight values and adjust the programming pulse width accordingly. So in this first section, we only take the sign of the gradient into account at each learning step and we apply identical pulses with width Δ*t* according to a simple heuristic, described in Alg. 1: whenever the desired weight change Δ*W* is positive (negative), we increase (decrease) *G*_+_ and decrease (increase) *G*_−_ by applying a pulse of duration Δ*t*. Note in Alg. 1 that the conductance update reads as ${G}_{ij}^{(n+1)}\leftarrow {G}_{ij}^{(n)}+{f}_{p,d}({G}_{ij}^{(n)},{\rm{\Delta }}t)$ with *f*_*p*_ and *f*_*d*_ respectively denoting the effective potentiation and depression occuring over a pulse width Δ*t* -see Methods for details.

**Algorithm 1:**
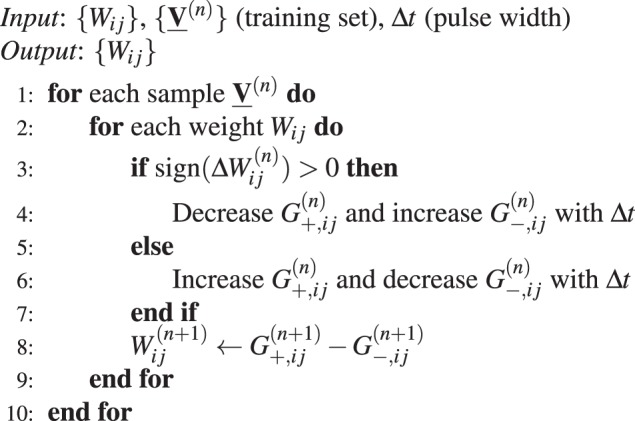
Memristor based gradient descent algorithm (**Cst**)

The pulse width monitors the speed of learning, and therefore has to be tuned. This algorithm has been called the Manhattan Rule^30^, Unregulated Step Descent^38^ or Stochastic Sign Descent (SSD)^39^. We herein call it Cst to refer to the fact that the programming pulse width is constant throughout learning.

In this work, we focus on the impact of non-linearity, cycle-to-cycle variability and device-to-device variability on the resulting performance, as illustrated in Fig. 1. Non-linearity is parametrized by *β* in Eq. (1), we modeled cycle-to-cycle variability by adding a Gaussian noise to each conductance update, and device-to-device variability by a dispersion on the multiplicative factor *C* appearing in Eq. (1) in which case one given device may not respond symmetrically to potentiation and depression, or devices of the same pair may not respond symmetrically to potentiation (see Methods).

### Resilience of RBM-based architectures trained with constant programming pulse width

A Restricted Boltzmann Machine (RBM) is a stochastic neural network, which learns to generate a data set. Physically, in such a network the neural dynamics are governed by an energy landscape. After learning, the minima of the energy have to correspond to the data set samples: neurons evolve towards a state that accounts for the data. The data is presented to visible units, denoted by *v* and the other neurons, called hidden neurons and denoted by *h*, are correlated to the visible units through the weights *W* and evolve accordingly. Visible and hidden units may also be influenced by a constant input which we model by a bias, respectively *b* and *c*. Formally, we can write the energy associated to such a system as:

4 $$E(v,h;W,b,c)=-\,\sum _{i,j}\,{W}_{ij}hi{v}_{j}-\sum _{i}\,{c}_{i}{h}_{i}-\sum _{j}\,{b}_{j}{v}_{j},$$

In practice, b and c are concatenated to W as an extra column and row respectively so that we absorb their definition into W without loss of generality. Neurons have binary values which are samples of the joint distribution $p(v,h)=\exp \,(\,-\,E(v,h))/{\sum }_{\tilde{v},\tilde{h}}\,\exp (\,-\,E(\tilde{v},\tilde{h}))$: running the neural dynamics amounts to sampling this distribution. Once the neural network is trained, such sampling is able to regenerate the data set. Learning is achieved by maximizing the log-likelihood *log p*(*v*) of the model on the data set whose exact rule reads:

5 $${\rm{\Delta }}{W}_{ij}={\langle {h}_{i}{v}_{j}\rangle }_{data}-{\langle {h}_{i}{v}_{j}\rangle }_{model},$$

where 〈.〉_*data*_ and 〈.〉_*model*_ denote a data average and a model average respectively. An estimate of this formula is provided by the approach of Constrastive Divergence^23^. The principle of this algorithm is to update the synaptic weights of the neural networks *W*_*ij*_ through:

6 $${\rm{\Delta }}{W}_{ij}={v}_{j}(0){h}_{i}(0)-{v}_{j}(1){h}_{i}(1)$$

States “0” and “1” refer to the step of a “Gibbs chain”, used to produce samples from the model. In step 0, the state of hidden neurons *h*(0) is sampled based on the state of input neurons *v*(0), clamped to a training example: *h*_*i*_(0) ~ *p*(*h*_*i*_|*v*_*j*_(0)) where ‘~’ means ‘is sampled from’. In step 1, the state of input neurons *v*(1) is sampled based on the previous state of hidden neurons (*v*_*j*_(1) ~ *p*(*v*_*j*_|*h*_*i*_(0))), and the state of the hidden neurons *h*(1) is sampled a second time based on the new state of the input neurons (*h*_*i*_(1) ~ *p*(*h*_*i*_|*v*_*j*_(1))). Note that *p*(*h*|*v*) = *σ*(*Wv*) and *p*(*v*|*h*) = *σ*(*W*^*T*^*h*) with *σ*(*x*) = 1/(1 + *exp*(−*x*)) so that the activation function used in a Restricted Boltzmann Machine is the usual sigmoid function. The most distinctive feature of Contrastive Divergence is its spatial locality. Unlike the backpropagation rule use for conventional forms of neural networks, the update to synaptic weight *W*_*ij*_ only depends on information about the two neurons *i* and *j* to which the synapse is connected.

**Figure 2:**
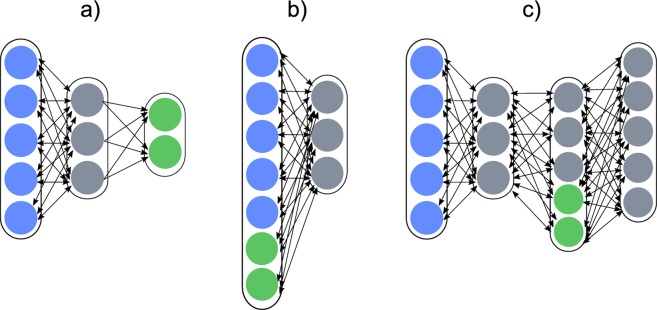
Schematics of the three architectures under study: (**a**) a Restricted Boltzmann Machine topped by a softmax classifier (“RBM + softmax”), (**b**) a Discriminative Restricted Boltzmann Machine (“Discriminative RBM”), (**c**) a Deep Belief Net.Blue, grey and green filled circles stand for visible, hidden and label neurons respectively.

The impact of RBM-based network topology has not been extensively investigated from a neuromorphic viewpoint^24,25,27^: a direct comparison of the influence of the position of the labels (i.e. placed in the visible layer or in a separate output layer) or of the depth of the network (i.e. stacking several RBMs) on the resulting performance with different device parameters has not yet been carried out. Our goal is to carry out such a comparison between different RBM-based architectures on the same learning task in terms of their resilience to device imperfections. We now present the results obtained when training under the Cst algorithm with typical device parameters the three most encountered RBM-based architectures in the neuromorphic literature on the MNIST discrimination task (see Table 1 and Fig. 2).

- The first one is a simple Restricted Boltzmann Machine (RBM) topped by a softmax classifier^24,27^ (“RBM + softmax”), with labels placed at the end of the network as the output of a classifier. In this architecture, the connections between input and hidden neurons, and output and hidden neurons are learned independently.
- The second is a Discriminative Restricted Boltzmann Machine^25,40^ taking as inputs both the picture and the associated label (“Discriminative RBM”). This architecture is expected to outperform the simple RBM, as the connections between input and hidden neurons, and output and hidden neurons are learned jointly.
- Finally, we simulate a Deep Belief Net consisting in a stack of two RBMs topped by a Discriminative RBM (“Deep Belief Net”, or DBN)^27,28^. As this architecture features three layers of hidden neurons, it is expected to be able to learn more difficult tasks than the other two architectures.

**Table 1:** Test error rate achieved by the three architectures under study on MNIST with typical values of the device parameters in terms of non-linearity, cyle-to-cycle and device-to-device variability.

Network topology		RBM + softmax	Discriminative RBM	Deep Belief Net
		785-301-10	795-301	785-501-511-2001
Software-based	Test error	7.0 ± 0.5%	6.6 ± 0.3%	5.7 ± 0.1%
Near-linear device *β* = 0.005	Test error	8.3 ± 0.1%	6.4 ± 0.2%	6.6 ± 0.2%
	$\frac{{\rm{\Delta }}{t}^{\ast }}{{\rm{\Delta }}{t}_{max}}$	$\frac{1}{1000}$	$\frac{1}{1000}$	$\frac{1}{150}$
Non-linear device *β* = 3	Test error	17.3 ± 0.3%	15 ± 0.1%	28.4 ± 0.3%
	$\frac{{\rm{\Delta }}{t}^{\ast }}{{\rm{\Delta }}{t}_{max}}$	$\frac{1}{5000}$	$\frac{1}{5000}$	$\frac{1}{10000}$
Cycle-to-cycle variability $\frac{{\sigma }_{intra}}{({G}_{max}-{G}_{min})}=6\cdot {10}^{-3}$, *β* = 0.005	Test error	15.1 ± 0.4%	11.9 ± 0.5%	9.2 ± 0.3%
Device-to-device variability ${(\frac{\sigma }{\mu })}_{inter}=1$, *β* = 0.005	Test error	20.3 ± 0.3%	13.9 ± 0.5%	22.6 ± 0.5%

Each topology includes the bias. Each simulation was performed over 30 epochs with a mini-batch size of 100, we indicate the mean error rate and the variance over five trials.

The three architectures are depicted on Fig. 2. A thorough description of the network hyperparameters and the methodology can be found in the Methods part. Throughout this paper, in contrast with most studies on the multi-layer perceptron, neurons are encoded with binary values at train and test time, and not real values. Moreover, as we restrict our study to local learning rules, the Deep Belief Net has only been trained two-layer wise as a stack of independent RBMs (i.e. “greedy learning”^41^), with no additional joint training with backpropagation (i.e. “fine-tuning”). Apart from the softmax classifier, all the architectures are trained using Contrastive Divergence. If not stated otherwise, the mini-batch size is set to 100.

Table 1 shows the mean optimal performance over five trials of the three networks on the test set with typical device parameters. For each set of device parameters, we tuned the pulse width Δ*t* until achieving the best performance: we denote the optimal pulse width for a given set of device parameters by Δt^*^. To make sense of our simulation results, we also performed floating point precision standard gradient descent simulation results, referred to as “software-based” in Table 1. In this situation, as an example, a Discriminative RBM achieves over five trials 6.6 ± 0.3% test error with 300 hidden units. This error rate can be reduced by the use of larger neural networks. With 500 hidden units, the Discriminative RBM achieves 5.4 ± 0.2%, and 3.6 ± 0.2% with 6000 hidden units which, up to the choice of hyperparameters, is akin to state-of-the-art for this type of architecture^22^.

**Figure 3:**
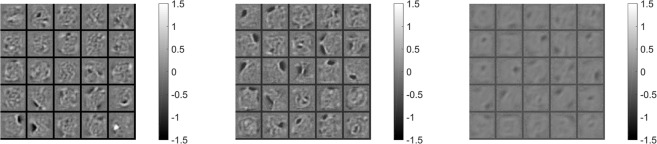
Examples of hidden features extracted by, from left to right: standard RBM (trained with a learning rate of 0.05), a memristive Discriminative RBM (trained under Cst with *β* = 0.005, Δ*t*/Δ*t*_*max*_ = 1/1000), another memristive Discriminative RBM (trained under Cst with *β* = 3, Δ*t*/Δ*t*_*max*_ = 1/5000). Each gray-scale picture represents the values of the 784 weights connecting the visible layer to a given hidden unit.

In this non-memristive floating point software-based training, the Deep Belief Net outperforms the other two networks, as one would expect. When using memristors, the near-linear case (*β* = 0.005) yields the best results for the three architectures compared to the non-linear case (*β* = 3), as it has been extensively observed on multi-layer perceptrons^12,17,42^. Interestingly, the Discriminative RBM achieves the lowest test error rate. It is not surprising that the RBM topped by a classifier may not do as well as the Discriminative RBM, as nothing ensures the features extracted by the RBM to be discriminative^22^. By contrast, it is surprising at first sight that the benefits of depth with the Deep Belief Net are not observed as in the floating point software-based training: the Deep Belief Net performs similarly to the Discriminative RBM when using near-linear memristors. However, the shape of the features accounts for these discrepancies. On Fig. 3, we display a 5 × 5 grid of gray-scale pictures, each of which representing the values of the 784 weights connecting the visible layer to a given hidden unit: each picture represents what is seen by one hidden unit, thus giving a direct insight into the features extracted by this hidden unit from the data. As seen per Fig. 3, while the features learned by a standard RBM (i.e. with a proper gradient descent) are sharply defined stroke-like features^43^, those learned by a memristive Discriminative RBM with the Cst algorithm are coarser. This may explain why stacking several memristive RBMs may not help for subtle features extraction and subsequently improved performance. This adds up to the fact we did not fine-tune the Deep Belief Net with backpropagation.

In the non-linear case (*β* = 3), the RBM topped by a softmax and the Discriminative RBM test error rates jumps by ~9% compared to ~22% for the Deep Belief Net. We can account for this observation with the corruption of the extracted features which, as seen from Fig. 3, is even more pronounced than in the linear case. As these corrupted features are fed into the next RBM, this effect cumulates with depth. When passing extracted features from one RBM to the next one, one stochastic realization may consequently not be enough to transmit all the information contained by the features. Finally, the pulse width for which the networks are tuned at *β* = 3 is lower than in the *β* = 0.005 case: non-linearity drags the optimal pulse width to low values to accommodate the abrupt conductance update it triggers.

When taking cycle-to-cycle variability into account in the linear case ($\frac{{\sigma }_{intra}}{({G}_{max}-{G}_{min})}=6\cdot {10}^{-3}$, *β* = 0.005), the Deep Belief Net appears to be more resilient to the programming noise than the two other networks: its test error rate only jumps by ~3% against ~6% for the two other networks. This happens because the pulse width for which the first two networks are tuned (Δt^*^/Δ*t*_*max*_ = 1/1000) is lower than the one of the Deep Belief Net (Δt^*^/Δ*t*_*max*_ = 1/150), so that the deterministic component of conductance update better dominates the programming noise. This idea will be further developed in the last section.

The impact of device-to-device variability in the linear case (${(\frac{\sigma }{\mu })}_{inter}=1$, *β* = 0.005) can also be interpreted in the light of the pulse width employed. As the coefficient carrying device-to-device variability comes in the memristor characteristic Eq. (1) as *C*_±_Δ*t*, the bigger Δ*t* the bigger the effect of device-to-device variability, which may explain why the Deep Belief Net is less resilient in this regard than the Discriminative RBM: the test error rate achieved by the latter increases by ~8% compared to ~16% for the former. Although the RBM topped by a softmax and the Discriminative RBM use the same pulse width, the former network turns out to be less resilient. The Discriminative RBM optimizes the joint probability of the inputs and labels so that device-to-device variability affects features extraction and classification consistently, which is not the case when RBM features are fed into an independent softmax classifier.

Overall, this comparative study reveals that the Discriminative RBM appears to be the best candidate architecture in terms of performance for typical values of the device parameters. We consequently focus our study in the rest of the paper on the Discriminative RBM. Still, the best performance for a given set of realistic device parameters is not satisfactory enough, and it is achieved for a very narrow range of pulse widths around the optimum. In the next two subsections, we propose two intuitive solutions to deal with these two aspects respectively, and finally combine them in the last subsection.

### Solutions mitigating device imperfections on the Discriminative RBM

#### Mitigating device non-linearity by reducing the variance of the gradient sign estimate

Gradient descent is inherently stochastic when dealing with a large data set. The first source of stochasticity comes from sampling a mini-batch of data drawn uniformly and independently from the data set and computing an approximate gradient over this mini-batch. A second source of stochasticity stems from Contrastive-Divergence itself, which relies on stochastic quantities, as seen in Eq. (6).

Most neuromorphic investigations on RBMs^24,25,27^ exacerbate these two forms of stochasticity, as Contrastive Divergence is carried out sample by sample (that is with a mini-batch of size one) using one single stochastic realization per neuron. In this section, we investigate techniques to reduce the stochasticity. First, we sum Eq. 6 across several samples (i.e. mini-batches). Second, we sum it over multiple stochastic realizations (i.e. parallel Gibbs chains). This second strategy amounts to encoding neurons by their firing rate instead of a single spike, and is reminiscent of the rate-coded Contrastive Divergence of^44^ or Event-driven Contrastive Divergence^45^.

**Figure 4:**
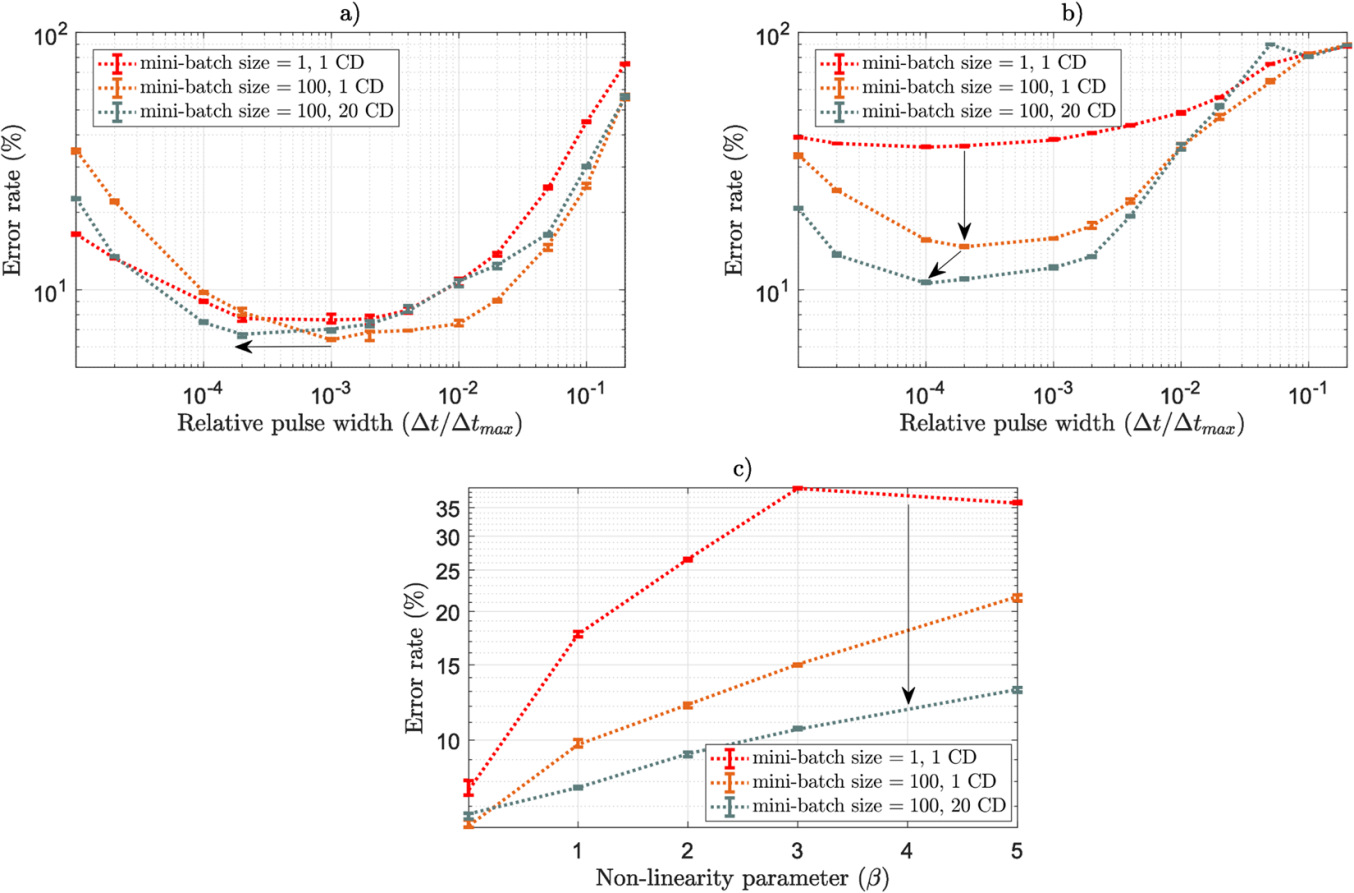
(**a**) Test error rate achieved by the Discriminative RBM as a function of the programming pulse width for different mini-batch sizes and different number of parallel Gibbs chains to evaluate the Contrastive Divergence term (‘# CD’) in the near-linear case (*β* = 0.005). (**b**) Same Figure as (**a**) in the non-linear case (*β* = 3). (**c**) Optimal test error rate achieved by the Discriminative RBM for different values of *β* with different mini-batch sizes and different number of parallel Gibbs chains to evaluate the Contrastive Divergence term (‘# CD’). For mini-batches size of 100, each simulation was ran over 30 epochs, 5 times per value of pulse width, error bars indicate median, first quartile and third quartile. For mini-batches of size 1, each simulation was ran over 50 epochs 5 times per value of pulse width to ensure convergence.

Figure 4(a,b) show the test error rate as a function of the pulse width used for the Discriminative RBM trained under the Cst algorithm, in the linear and non-linear case, and with different mini-batch sizes and numbers of parallel Gibbs chains used for Contrastive Divergence. In the linear case (Fig. 4a)), increasing the mini-batch size or the number of parallel Gibbs chains does not improve significantly the resulting performance. Conversely, when working with non-linear devices (Fig. 4b)), decreasing the variance of the gradient estimate dramatically makes a difference. Decreasing the variance of the gradient estimate indeed helps the conductances to move into good directions, especially when the conductance increment is abrupt and uncontrolled in the non-linear case. Moreover, the optimal pulse width is dragged towards smaller values when decreasing the variance of the sign of the gradient estimate: with a reduced variance and within a fixed number of epochs, the algorithm converges faster and subsequently selects a smaller learning rate. This could seem counter-intuitive, as in a standard gradient descent framework, the optimal mini-batch size is known to scale linearly with the learning rate^46^. However, this analysis does not hold here upon only taking the sign of the gradient into account. Figure 4(c) shows for each value of *β* the best error rate achieved when using one or 20 parallel Gibbs chains, both with a mini-batch size of 100, supporting the above statement.

#### Facilitate pulse width tuning: Resilient Propagation (RProp)

Using a constant pulse width may not be optimal for several reasons. As the amplitude of the weight updates is directly monitored by the programming pulse width and amplitude, it has to be tuned with a hyperparameter selection by sweeping through different values. Also, when using identical pulses throughout learning, undesirably large weight updates may occur in conductance regions of high non-linearity, entailing weight dithering around optima^12^. Conversely with a pulse width that is too small, conductances may move too slowly for convergence to be achieved within a reasonable number of epochs. A natural solution is to drop the Manhattan rule by reading out the numerical value of the gradient itself and applying the number of pulses required^12,17,47^, or emulating linearity with pulses consistent with the current conductance state^48,49^. However, these solutions are very expensive in practice: they require reading the state of each memory device at each learning update. Here, we investigate a simpler to implement solution, which exploits information about neurons only. In a system, informations about neurons is indeed much more readily available than information about the memory devices.

Interestingly, off-line conductance tuning protocols, gradually increasing the pulse width or voltage amplitude so long as we get closer to a conductance target or decreasing it otherwise^7,10^, give some insights into appropriate on-line programming schemes. A mathematical generalization of this heuristic, consisting in increasing the learning rate so long as we get closer to an optimal model or decreasing it otherwise, is called Resilient Propagation^30–32^ (RProp). Very recently, a RProp-like technique was proposed for training a memristive multi-layer perceptron and was named the “Local Gain Techniques”^50^. In this work, we take inspiration in an improved version of RProp with “weight back-tracking” (called RProp+ in^31^), which cancels conductance updates that overshot an optimal model, and subsequently reduces the pulse width.

**Figure 5:**
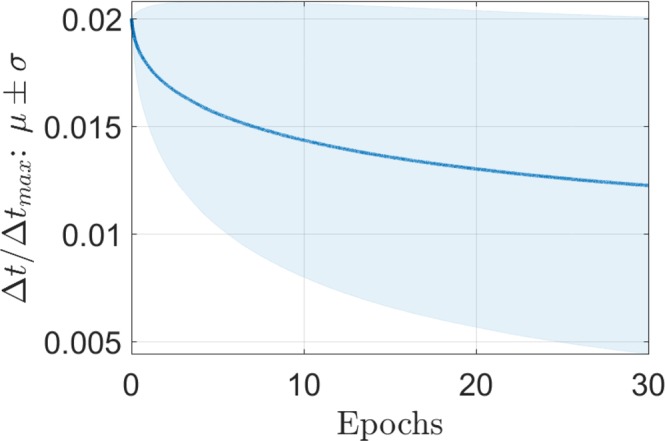
Typical time trace of the Δ*t*_*ij*_/Δ*t*_*max*_ statistics in terms of the mean value (blue plain line) and standard deviation (shaded blue region around the line).

A detailed description of the neuromorphic adaptation of RProp with weight back-tracking is presented in Alg. 2. Whenever the sign of the gradient remains the same, the pulse width is increased by a factor *η*_+_ > 1 so long as it does not exceed the initial pulse width: Δ*t*_*ij*_ ← *min*(*η*_+_Δ*t*_*ij*_, Δ*t*(0)). This condition emulates a learning rate decay from its initial value, as seen per Fig. 5. When a gradient sign flip is encountered, we cancel the last conductance change over the same pulse width, and decrease the pulse width for the next learning step by a factor *η*_−_ < 1. Note from Alg. 2 that we did not impose a minimal pulse width, we allow it to decay to zero. By construction, the pulse width is consequently bounded by initial pulse width and zero: Δ*t*_*max*_ = Δ*t*(0), Δ*t*_*min*_ = 0. This weight-backtracking is meant to avoid penalizing twice the algorithm by overshooting a local optimum and not going back far enough to cancel the wrong conductance move, and is handled by the third logic case Δ*W*^(*n*)^Δ*W*^(*n*+1)^ = 0 (see Alg. 2). In addition, the pulse width is bounded by the initial pulse width.

**Algorithm 2:**
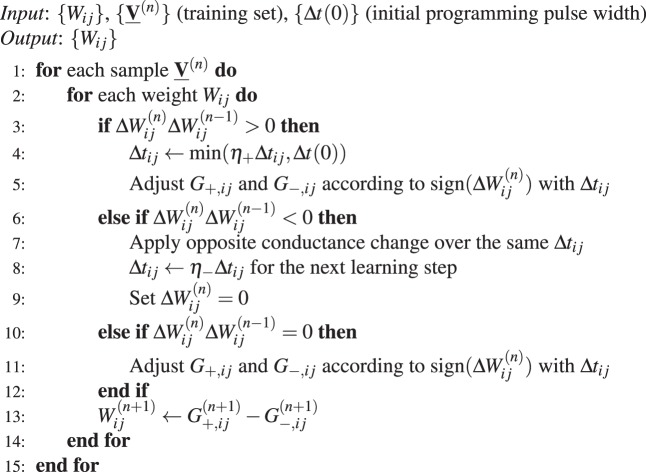
Memristor based gradient descent algorithm (**RProp**)

As seen in Table 2, in spite of the apparent complexity of the RProp, it can be handled easily when applied to Contrastive Divergence. In Table 2, we denote ${N}_{+,ij}^{(n)}$ the positive term of Contrastive Divergence, i.e. *v*_*j*_(0)*h*_*i*_(0), and ${N}_{+,ij}^{(n)}$ the negative term, i.e. *v*_*j*_(1)*h*_*i*_(1)), summed across mini-batches and parallel Gibbs chains. The relative importance of *N*_+,*ij*_ and *N*_+,*ij*_ between two consecutive learning steps *n* − 1 and *n* can be classified in the nine logic cases depicted in Table 2. From these nine cases, we can deduce the sign of the factor $\Delta {W}_{ij}^{(n-\mathrm{1)}}\Delta {W}_{ij}^{(n)}$, which is essential for RProp. This shows that our RProp-type rule can be implemented with knowledge about the neurons only, and relatively simple logics.

**Table 2:** RProp table of truth for any mini-batch size and number of parallel Gibbs chains. The notations are defined on the body text.

$$\Delta {W}_{ij}^{(n)}={N}_{+,ij}^{(n)}-{N}_{-,ij}^{(n)}$$					
*n* − 1	${N}_{+,ij}^{(n-\mathrm{1)}} > {N}_{-,ij}^{(n-1)}$	${N}_{+,ij}^{(n-1)} < {N}_{-,ij}^{(n-1)}$	${N}_{+,ij}^{(n-1)} > {N}_{-,ij}^{(n-1)}$	${N}_{+,ij}^{(n-\mathrm{1)}} < {N}_{-,ij}^{(n-1)}$	5 other cases
*n*	${N}_{+,ij}^{(n)} > {N}_{-,ij}^{(n)}$	${N}_{+,ij}^{(n)} < {N}_{-,ij}^{(n)}$	${N}_{+,ij}^{(n)} < {N}_{-,ij}^{(n)}$	${N}_{+,ij}^{(n)} > {N}_{-,ij}^{(n)}$	
Case	${\rm{\Delta }}{W}_{ij}^{(n-\mathrm{1)}}{\rm{\Delta }}{W}_{ij}^{(n)} > 0$		${\rm{\Delta }}{W}_{ij}^{(n-\mathrm{1)}}{\rm{\Delta }}{W}_{ij}^{(n)} < 0$		${\rm{\Delta }}{W}_{ij}^{(n-1)}{\rm{\Delta }}{W}_{ij}^{(n)}=0$

**Figure 6:**
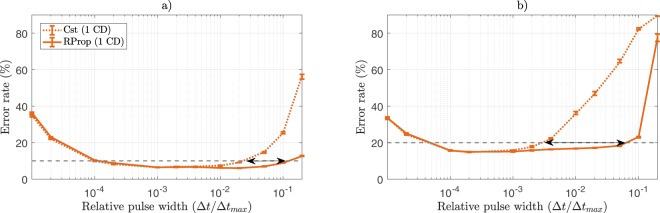
(**a**) Test error rate achieved by the Discriminative RBM as a function of the programming pulse width when trained with Cst and RProp driven pulse widths for *β* = 0.005. (**b**) Same as (**a**) with *β* = 3. Grey dashed lines indicate 10% and 20% on the left and right panel respectively. Each simulation was ran over 30 epochs with a mini-batch size of 100, 5 times per value of pulse width, error bars indicate median, first quartile and third quartile.

Figure 6 shows the comparative performance of the Discriminative RBM trained with Cst and our RProp rule, for varying initial pulse widths. In the linear case (*β* = 0.005), RProp allows achieving a test error that is lower than 10% for ${\rm{\Delta }}t/{\rm{\Delta }}{t}_{max}\in {\mathrm{[10}}^{-4}, \sim \,{10}^{-1}]$, compared to ${\rm{\Delta }}t/{\rm{\Delta }}{t}_{max}\in {\mathrm{[10}}^{-4}, \sim \,{2.10}^{-2}]$ when using the Cst algorithm. Similarly in the non-linear case (*β* = 3), RProp allows achieving a test error that is lower than 20% for ${\rm{\Delta }}t/{\rm{\Delta }}{t}_{max}\in [ \sim {5.10}^{-5}, \sim \,{7.10}^{-2}]$, compared to ${\rm{\Delta }}t/{\rm{\Delta }}{t}_{max}\in [ \sim {5.10}^{-5}, \sim \,{3.10}^{-3}]$ when using the Cst algorithm. In this regard, RProp manages to extend the range of eligible pulse widths.

#### Resilience to cycle-to-cycle variability

In this section, we investigate the resilience of the Discriminative RBM to cycle-to-cycle variability and device-to-device variability using the two techniques introduced above. We restrict our study to the linear case (*β* = 0.005) to ensure that our results are not biased by non linearity.

**Figure 7:**
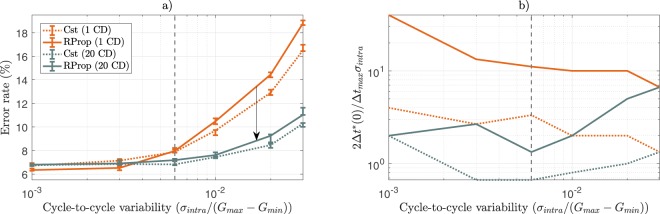
(**a**) Test error rate achieved by the Discriminative RBM as a function of cycle-to-cycle variability for every combination of the pulse width programming scheme (Cst, RProp) and number of parallel Gibbs chains used to evaluate Contrastive Divergence (# CD). (**b**) Optimal conductance increment-to-noise ratio as a function of cycle-to-cycle variability associated with each curve of the left panel. When using 20 Gibbs chains (blue curves), from *σ*_*intra*_/(*G*_*max*_ − *G*_*min*_) = 6.10^−3^ onwards (vertical gray dashed line) the conductance update overcomes the noise increase, accounting for the improved performance compared to the use of a single Gibbs chain (orange curves), regardless of the programming scheme. Each simulation was ran over 30 epochs with a mini-batch size of 100, 5 times per value of pulse width, error bars indicate median, first quartile and third quartile.

We present in Fig. 7(a) the impact of cycle-to-cycle variability upon the performance of the Discriminative RBM trained under the four possible combinations of the training techniques studied before. As mentioned above, using longer programming pulses may be preferable in the presence of cycle-to-cycle variability. Therefore, we tuned learning for the best pulse width for each given noise intensity.

We first observe that using Cst or RProp only weakly changes the resilience to cycle-to-cycle variability. By contrast, using multiple Gibbs chains improves the performance by ~6% with the maximal amount of cycle-to-cycle variability. This result might seem initially surprising, as the systems with 20 Gibbs chains are tuned at a smaller pulse width than the systems with one Gibbs chain at low noise, thus intuitively more sensitive to noise.

To facilitate the cycle-to-cycle variability analysis, we present the conductance increment-to-noise ratio in the linear case 2*C*Δt^*^(0)/(*σ*_*intra*_(*G*_*max*_ − *G*_*min*_)) = 2Δ*t*(0)/(Δ*t*_*max*_*σ*_*intra*_), computed for each level of noise and optimal pulse width Δt^*^(0), in Fig. 7(b). When using a single Gibbs chain, the conductance increment-to-noise ratio steadily decreases when noise increases: the noise increase dominates the conductance update. By contrast, when using 20 Gibbs chains, this parameter increases with noise from *σ*_*intra*_/(*G*_*max*_ − *G*_*min*_) = 6.10^−3^ onwards: the conductance update starts to overcome the noise increase. This value corresponds to the level of noise for which a clear difference appears between on and 20 Gibbs chains in Fig. 7(a), so that our analysis in terms of the conductance increment-to-noise ratio relevantly accounts for this discrepancy.

All of these observations boil down to how much the pulse width can be increased to absorb noise: what matters is not the pulse width for which the algorithms are tuned in the absence of noise, but how much it can be increased from there to absorb noise. Consequently, it is precisely because the systems using 20 Gibbs chains are tuned at a smaller pulse width than (Cst, 1 CD), and itself smaller than (RProp, 1 CD) that the former are more resilient to noise than the latter.

#### Resilience to device-to-device variability

**Figure 8:**
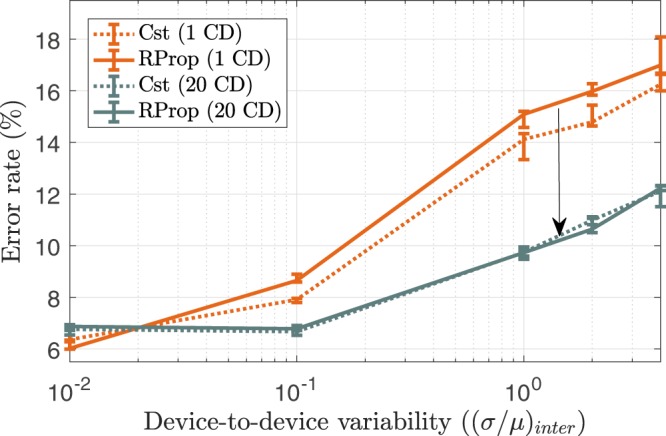
Test error rate achieved by the Discriminative RBM as a function of device-to-device variability for every combination of the pulse width programming scheme (Cst, RProp) and the number of parallel Gibbs chains (# CD). Each simulation was ran over 30 epochs with a mini-batch size of 100, 5 times per value of pulse width, error bars indicate median, first quartile and third quartile.

While analyzing the impact of cycle-to-cycle variability on the performance involves many phenomena, understanding the impact of device-to-device variability for the four different schemes is straightforward and similar to the analysis carried out in Table 1: the larger the pulse width, the bigger the impact of device-to-device variability on the performance. Figure 8 shows that the schemes using 20 parallel Gibbs chains are more robust to device-to-device variability than the scheme with Cst driven pulse widths and 1 Gibbs chain, itself more robust than its RProp counterpart, which is directly accounted by their respective pulse widths. Table 3 summarizes the results obtained with all the possible combinations of the techniques used and for typical values of the device parameters.

**Table 3:** Summary of the results obtained on the Discriminative RBM.

		1 CD, Cst	1 CD, RProp	20 CD, Cst	20 CD, RProp
Near-linear device *β* = 0.005	Test error	6.4 ± 0.1%	6 ± 0.2%	6.7 ± 0.1%	6.7 ± 0.2%
	$\frac{{\rm{\Delta }}{t}^{\ast }}{{\rm{\Delta }}{t}_{max}}$	$\frac{1}{1000}$	$\frac{1}{50}$	$\frac{1}{5000}$	$\frac{1}{5000}$
	$\frac{{\rm{\Delta }}t}{{\rm{\Delta }}{t}_{max}}$ s.t. Test error < 10%	[10^−4^, 2.10^−2^]	[10^−4^, 10^−1^]	[5.10^−5^, 10^−2^]	[5.10^−5^, 5.10^−2^]
Non-linear device *β* = 3	Test error	14.7 ± 0.2%	14.9 ± 0.2%	10.7 ± 0.2%	10.5 ± 0.2%
	$\frac{{\rm{\Delta }}{t}^{\ast }}{{\rm{\Delta }}{t}_{max}}$	$\frac{1}{5000}$	$\frac{1}{5000}$	$\frac{1}{10000}$	$\frac{1}{10000}$
	$\frac{{\rm{\Delta }}t}{{\rm{\Delta }}{t}_{max}}$ s.t. Test error < 20%	[5.10^−5^, 3.10^−3^]	[5.10^−5^, 7.10^−2^]	[10^−5^, 4.10^−3^]	[10^−5^, 2.10^−2^]
Cycle-to-cycle variability $\frac{{\sigma }_{intra}}{({G}_{max}-{G}_{min})}=6\cdot {10}^{-3}$, *β* = 0.005	Test error	16.8 ± 0.7%	18.8 ± 0.3%	10.2 ± 0.2%	11.2 ± 0.1%
Device-to-device variability ${(\frac{\sigma }{\mu })}_{inter}=1$, *β* = 0.005	Test error	13.9 ± 0.6%	14.9 ± 0.4%	9.6 ± 0.3%	10.8 ± 0.3%

Each simulation was ran over 30 epochs with a mini-batch size of 100, 5 times per value of pulse width, error bars indicate median, first quartile and third quartile.

## Discussion

To design hardware-friendly learning rules that are both local and resilient to imprecise programming of memristive devices, we first studied the three most encountered RBM-based neural networks in the neuromorphic literature in terms of their performance on the MNIST discrimination task, when trained under our baseline memristor-based gradient descent algorithm (Cst). With typical values of non-linearity, cycle-to-cycle and device-to-device variabilities, the Discriminative RBM outperforms the two other architectures. Using one bit of information at each learning step (i.e. the sign of the gradient) with one bit per neuron (i.e. stochastically sampled binary neurons) while achieving a classification performance akin to software-based simulations, the Discriminative RBM trained under Contrastive Divergence appears to be a good candidate for *in situ* learning. Also, the choice of the pulse width is critical with respect to the device imperfections. While hand-tuning the programming width as a hyperparameter selects an optimal value that is never predictable in advance, we can understand how the weight update physics influence it. Increasing non-linearity or device-to-device variability, with regards to an ideal device, favors pulse widths that are shorter to avoid abrupt conductance changes. Conversely increasing cycle-to-cycle variability selects pulse widths that are longer to overshadow the programming noise with respect to the amplitude of the conductance update.

More importantly, and surprisingly at first sight, the Deep Belief Net does not perform better than the Discriminative RBM. On the one hand, the inefficiency of depth in our specific training and inference setting is due to the coarsened feature extraction abilities of RBMs upon using memristive devices. In the best case (near-linear) the stack of RBMs is not useful, in the worst case (non-linear), learning is dramatically jeopardized when passing corrupted features into downstream RBMs. On the other hand, this inefficiency also stems from not fine-tuning the stack of RBMs with backpropagation, as per our choice to solely focus on local learning rules.

For advanced applications, Discriminative RBMs could nonetheless be used within deep neural networks to learn complex tasks, if the transfer learning approach is used. This approach consists in importing upstreams weights previously trained on software for feature extraction on a particular kind of data, and training *in situ* only the last layers on similar but more specific data^6^. This strategy has been proven in various contexts, and allows training neural networks on new tasks with relatively modest amounts of data, if the neural network has been previously trained on a different but yet similar task with important amounts of data^51^. Therefore, our simplified technique for training should not be considered for training deep neural networks in their entirety, but for adapting them to new situations, for example in embedded environments.

In the second part of this work, we showed that decreasing the variance of Contrastive Divergence by summing it across samples (i.e. mini-batches) and stochastic realizations of neurons (i.e. parallel Gibbs chains) considerably improved the performance of Discriminative RBMs, when trained with realistic memristive devices. This trend was seen in terms of non-linearity, cycle-to-cycle and device-to-device variability. Decreasing the variance of Contrastive Divergence indeed makes the algorithm more immune to non-linearity: it is no longer penalized by abrupt conductance changes along wrong directions. And as this technique selects a smaller programming pulse width, it can smoothen out the discrepancies due to variability sources. Interestingly, our findings on the Discriminative RBM shed new light on the impact of the device imperfections by entangling them all around the choice of the programming pulse width, which has to be tediously tuned when it is fixed throughout learning (Cst). This statement pushed us to investigate the use of RProp driven pulse widths. By taking into account the sign of the gradient between two consecutive learning steps, this technique enables to enlarge the range of sensible pulse widths by up to two decades without affecting the resilience to the device imperfections. From Figs 7, 8 and Table 3, we acknowledge that the use RProp may not always yield the best error rate when tuned at its optimal pulse width. However, we see from Fig. 6 that RProp outperforms Cst when taking the whole range of pulse widths into account. Also, while we explicitly studied the combination of RProp with the use of multiple Gibbs chains with regards to variability sources, we want to stress here its impact upon non-linearity effects as it appears in Table 3: (20 CD, Cst) and (20 CD, RProp) at *β* = 3 achieve the same optimal performance and the only effect of RProp is to enlarge the range of pulse widths achieving a test error that is lower than 20%. Thus this technique is of definite practical interest as it reduces the need to tune hyperparameters, a major concern for learning in embedded contexts.

Our choices regarding device modeling were guided by the existing literature. For instance, putting device-to-device variability into the multiplicative parameter *C* appearing in Eq. (1) is inspired by device measurements^35^. Our work would not apply directly to Phase Change Memory devices which exhibit a strong asymmetry between potentiation and depression. However, our results would be applicable directly to pairs of Phase Change Memories associated in 2-PCM structures^12^. We also bear in mind that the use of mini-batches calls for the design of elaborate memory devices. A promising path to accomplish on-chip mini-batch gradient descent could be inspired by recent works^6^, combining a volatile and a non-volatile memory that would be updated within and across mini-batches respectively.

Overall, these results suggest the possibility to achieve on chip learning with memristive learning with Discriminative Restricted Boltzmann machines, using a local learning rule and very simple device programming, and highlights strategies to make the learning process viable even with highly imperfect devices. More generally, our results highlight that the methods for making learning functional with imperfect analog hardware can differ from the techniques used for standard machine learning in software, suggesting the high need for hardware and learning algorithm codevelopment.

## Methods

### Memristor model used

Integrating Eq. (1) between *t*_0_ and *t*_0_ + Δ*t* yields the explicit effective conductance update:

7 $$\begin{array}{ccc}G({t}_{0}+{\rm{\Delta }}t)-G({t}_{0}) & = & \{\begin{array}{cc}{f}_{p}(G({t}_{0}),{\rm{\Delta }}t)=\frac{{G}_{max}-{G}_{min}}{{\beta }_{p}}\,{\rm{l}}{\rm{o}}{\rm{g}}(1+{\textstyle \tfrac{{\beta }_{p}}{{G}_{max}-{G}_{min}}}{C}_{p}{\rm{\Delta }}t\,\exp \,(\,-\,{\beta }_{p}{\textstyle \tfrac{G({t}_{0})-{G}_{min}}{{G}_{max}-{G}_{min}}})) & ({\rm{p}}{\rm{o}}{\rm{t}}{\rm{e}}{\rm{n}}{\rm{t}}{\rm{i}}{\rm{a}}{\rm{t}}{\rm{i}}{\rm{o}}{\rm{n}})\\ {f}_{m}(G({t}_{0}),{\rm{\Delta }}t)=-{\textstyle \tfrac{{G}_{max}-{G}_{min}}{{\beta }_{d}}}log(1+\frac{{\beta }_{d}}{{G}_{max}-{G}_{min}}{C}_{d}{\rm{\Delta }}t\,\exp \,(\,-\,{\beta }_{d}{\textstyle \tfrac{{G}_{max}-G({t}_{0})}{{G}_{max}-{G}_{min}}})) & ({\rm{d}}{\rm{e}}{\rm{p}}{\rm{r}}{\rm{e}}{\rm{s}}{\rm{s}}{\rm{i}}{\rm{o}}{\rm{n}}),\end{array}\end{array}$$

Equation (7) is the explicit form of Eq. (2). In most of our simulations, we took *C*_*p*_ = *C*_*d*_ = *C* and *β*_*p*_ = *β*_*d*_ = *β*. The single part of the study where this symmetry is broken is when studying device-to-device variability -see below. The constant C, encoding the voltage amplitude applied to the device, is fixed by the condition Δ*G*(*t*_0_, Δ*t*_*max*_) = *G*_*max*_ − *G*_*min*_, *G*(*t*_0_) = *G*_*min*_ yielding with Eq. (7) $C=\frac{{G}_{max}-{G}_{min}}{{\rm{\Delta }}{t}_{max}}\frac{\exp \,\beta -1}{\beta }$. Injecting this C back into Eq.(7) shows that only the ratio Δ*t*/Δ*t*_*max*_ is relevant. Note that *C* depends on *β* so that whenever *β* was changed, so was *C*. In all the simulations, *G*_*max*_ was taken to be 1 and *G*_*min*_ = 1/13. To model cycle-to-cycle variability, we simply added a Gaussian noise to each conductance update dictated by Eq.(7), e.g. Δ*G*_*tot*_(*t*_0_, Δ*t*) = Δ*G*(*t*_0_, Δ*t*)+ *noise* with noise $\sim \,{\mathscr{N}}(0,{\sigma }_{intra}^{2})$ with *σ*_*intra*_ = *ε*_*intra*_(*G*_*max*_ − *G*_*min*_). The parameter *ε*_*intra*_ is the actual quantity we called ‘cycle-to-cycle variability’ throughout the paper: its value was swept through {0.001, 0.003, 0.006, 0.01, 0.02, 0.03}. For a given *β*, device-to-device variability was modeled by adding a dispersion on the coefficient *C* with

$C \sim \,\mathrm{log}\,{\mathscr{N}}(\mathrm{log}\,\frac{\bar{C}}{\sqrt{1+{\varepsilon }_{inter}^{2}}},\sqrt{\mathrm{log}(1+{\varepsilon }_{inter}^{2})})$ so that $\langle C\rangle =\bar{C}$ $\sigma (C)={\varepsilon }_{inter}\bar{C}$ with $\bar{C}=\frac{{G}_{max}-{G}_{min}}{{\rm{\Delta }}{t}_{max}}\frac{\exp \,\beta -1}{\beta }$. The parameter *ε*_*inter*_ is the actual quantity we called ‘device-to-device variability’ throughout the paper: its value was swept through {0.01, 0.1, 1, 2, 4}. Note that consequently in this very particular case: *C*_+,*p*_ ≠ *C*_+, *p*_ (one given device do not respond symmetrically to potentiation and depression) and *C*_+,*p*_ ≠ *C*_−, *p*_ (devices of the same pair do not respond symmetrically to potentiation).

Finally note that our model defined as Eq. (1) can look similar to^36^:

8 $$\frac{dG(t)}{dt}=\beta (G(t),n,{\rm{\Delta }}V(t),{\rm{\Delta }}t)(1-\frac{G(t)}{{G}_{max}}),$$

with some important differences however. The coefficient *β* does not have the same meaning in the two models: while it is a constant in ours, it appears in theirs as a function of the applied voltage height (Δ*V*(*t*)) and width (Δ*t*) to model switching dynamics which we did not take into account. Morever, the number of pulses applied (*n*) appears explicitly in their model while it is implicit in ours: we treat equally a pulse of length Δ*t* and *n* pulses of length Δ*t*/*n*. Finally, the dependence of the conductance update with the current conductance is exponential in our model while it is polynomial in theirs. Expanding Eq. (1) for a small *β* and assuming *G*_*max*_ >> *G*_*min*_ brings our model the closest to Eq. (8), up to the linear contribution in *C*:

9 $$\frac{dG(t)}{dt}\, \sim {}_{\beta \to 0}\{\begin{array}{ll}C+\beta \frac{C{G}_{min}}{{G}_{max}}(1-\frac{G(t)}{{G}_{min}}) & ({\rm{potentiation}})\\ -C+\beta C(1-\frac{G(t)}{{G}_{max}}) & ({\rm{depression}})\end{array}.\,$$

### Simulations

All the simulations presented in this work have been carried out in the most simple way in the sense that it is abstracted from the realistic constraints inherent to the crossbar circuitry. We assumed that the memristive devices are associated with an access device (transistor^6^ or resistive switching selector device^52,53^), and therefore neglected sneak paths current effects. Sneak paths currents, if present, would significantly decrease the accuracy in programming the synaptic weights. Our goal is to focus on the effects of the weight update physics and the learning rules it enables on the different neural network architectures introduced above, so as to motivate further realistic investigations.

When training any architecture throughout the paper, we used 40,000 samples for training and 10,000 for test from the MNIST data base. The different neural network topologies (bias included) were set as follows: 785-301-10 for the RBM + softmax stack, 794(784 + 10) + 300 for the Discriminative RBM and 785 + 501 + 511(501 + 10) + 2001 for the Deep Belief Net. In practice, biases were concatenated to W as an extra column and row. Labels are one-hot encoded, e.g. the label “2” is encoded as (0, 1, 0, 0, 0, 0, 0, 0, 0, 0) out of 10 possible outcomes. If not stated otherwise, all simulations were carried out with a mini-batch size of 100.

The benchmark floating point software-based simulation results on the Discriminative with 300, 500 and 6000 hidden units have been obtained in specific training and test conditions apart from the memristor-based simulations. During training when computing Contrastive Divergence, visible units are binarized while hidden units are encoded by probabilities as it has been empirically prescribed^54^. Only one step of Gibbs sampling was used to compute Contrastive Divergence (CD-1). At test time and contrary to memristor-based simulations, the inference technique is deterministic: when clamping a digit the network was not trained on, the label which is selected is the one which minimizes the free-energy *F*(*v*) of the RBM:

10 $$F(v)=-\,\mathrm{log}\,\sum _{h}\,\exp \,({h}^{T}Wv),$$

with $p(v)=\exp \,(\,-\,F(v))/{\sum }_{\tilde{v}}\,\exp \,(-\,F(\tilde{v}))$: a minimal free-energy corresponds to a maximal likelihood. The Discriminative RBMs with 300, 500 and 6000 hidden units have respectively been tuned with a learning rate of 0.01, 0.01 and 0.04.

We now specify memristor-based simulation conditions. As in^13^, the conductance were sampled from

${\mathscr{N}}\,(\frac{{G}_{max}-{G}_{min}}{2},{(0.1\frac{{G}_{max}-{G}_{min}}{2})}^{2})$ and the conductance bias were set to $\frac{{G}_{max}-{G}_{min}}{2}$. In any architecture, the neurons values during inference (i.e. during the forward pass at a given training step) and training (i.e. in the weight update itself) were binarized, i.e. stochastically sampled from their Bernoulli probability given their upstream neurons, using sigmoid activation functions. When greedily training the Deep Belief Net, we trained the 785 + 501 RBM taking as inputs binarized MNIST inputs, then the 501 + 501 RBM taking as inputs binarized features extracted by the first RBM, finally the 511 + 2001 Discriminative RBM taking as inputs features extracted by the second RBM and target labels. To perform inference in the Discriminative RBM on the label units to calculate the test error rate (this is strictly analogous in the Deep Belief Net, taking extracted features instead of the MNIST samples as inputs), we proceeded in the following way: we initially clamp a given test sample on the first 784 units (or extracted features of this test sample on the first 500 hidden units) along with a label vector on the remaining 10 units initialized to (1/10)(1,…,1). We subsequently perform 40 Gibbs chains in parallel over 2 steps (exactly as in^45^) and average the resulting 40 label vectors to determine which label was selected by the network.

If not stated otherwise, simulations were performed over 30 epochs, over 5 trials, with error bars indicating median, first quartile, third quartile with a mini-batch size of 100.

Finally, the multiplicative coefficient *η*_+_ appearing in Alg. (2) describing RProp was set empirically set to 1.01 and we fixed *η*_−_ = 1/*η*_+_. We selected *η*_+_ by simply drawing the cumulative distribution function of the final pulse widths and ensured that it was spread enough over the whole range [0, Δ*t*_*max*_] - with *η* = 1.05 conversely, 40% of the device were shut off after 30 epochs of learning (Δ*t* = 0).

All simulation scripts can be found on: https://github.com/ernoult/mem-RBM.git.

## Data Availability

The datasets generated analysed during the current study are available from the GitHub repository: https://github.com/ernoult/mem-RBM/tree/master/git-hub/RESULTS_PAPER.
